# Supplementary material for: Evaluating mesothelin as an immunotherapeutic target for endogenous T cells
Source: Front Immunol. 2026 May 29;17:1853409. doi: 10.3389/fimmu.2026.1853409 (PMC13260466; doi:10.3389/fimmu.2026.1853409)
Supplement: Supplementary file 1 [file Presentation1.pptx]

## Slide 1
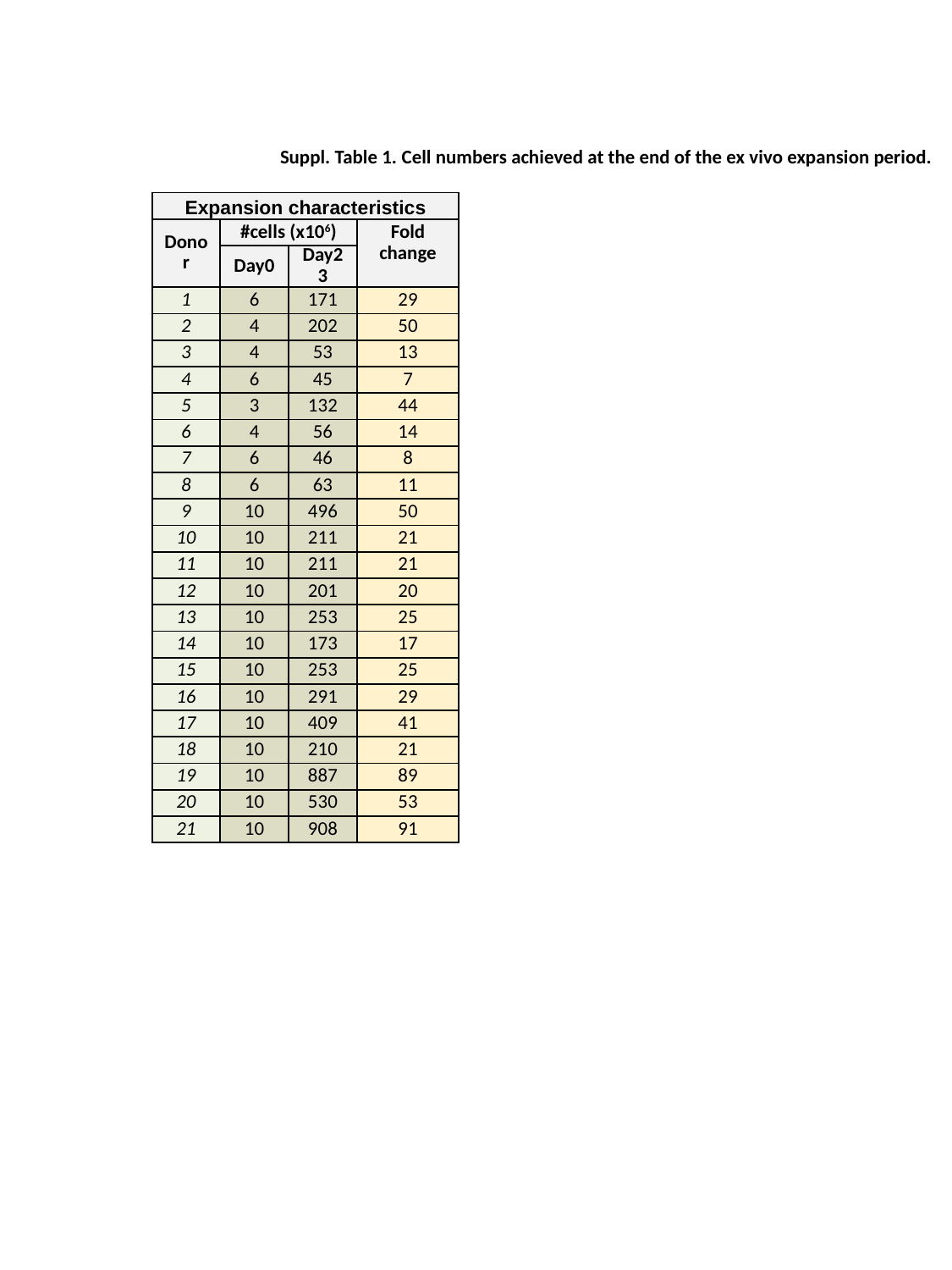

Suppl. Table 1. Cell numbers achieved at the end of the ex vivo expansion period.
| Expansion characteristics | | | |
| --- | --- | --- | --- |
| Donor | #cells (x106) | | Fold change |
| | Day0 | Day23 | |
| 1 | 6 | 171 | 29 |
| 2 | 4 | 202 | 50 |
| 3 | 4 | 53 | 13 |
| 4 | 6 | 45 | 7 |
| 5 | 3 | 132 | 44 |
| 6 | 4 | 56 | 14 |
| 7 | 6 | 46 | 8 |
| 8 | 6 | 63 | 11 |
| 9 | 10 | 496 | 50 |
| 10 | 10 | 211 | 21 |
| 11 | 10 | 211 | 21 |
| 12 | 10 | 201 | 20 |
| 13 | 10 | 253 | 25 |
| 14 | 10 | 173 | 17 |
| 15 | 10 | 253 | 25 |
| 16 | 10 | 291 | 29 |
| 17 | 10 | 409 | 41 |
| 18 | 10 | 210 | 21 |
| 19 | 10 | 887 | 89 |
| 20 | 10 | 530 | 53 |
| 21 | 10 | 908 | 91 |

## Slide 2
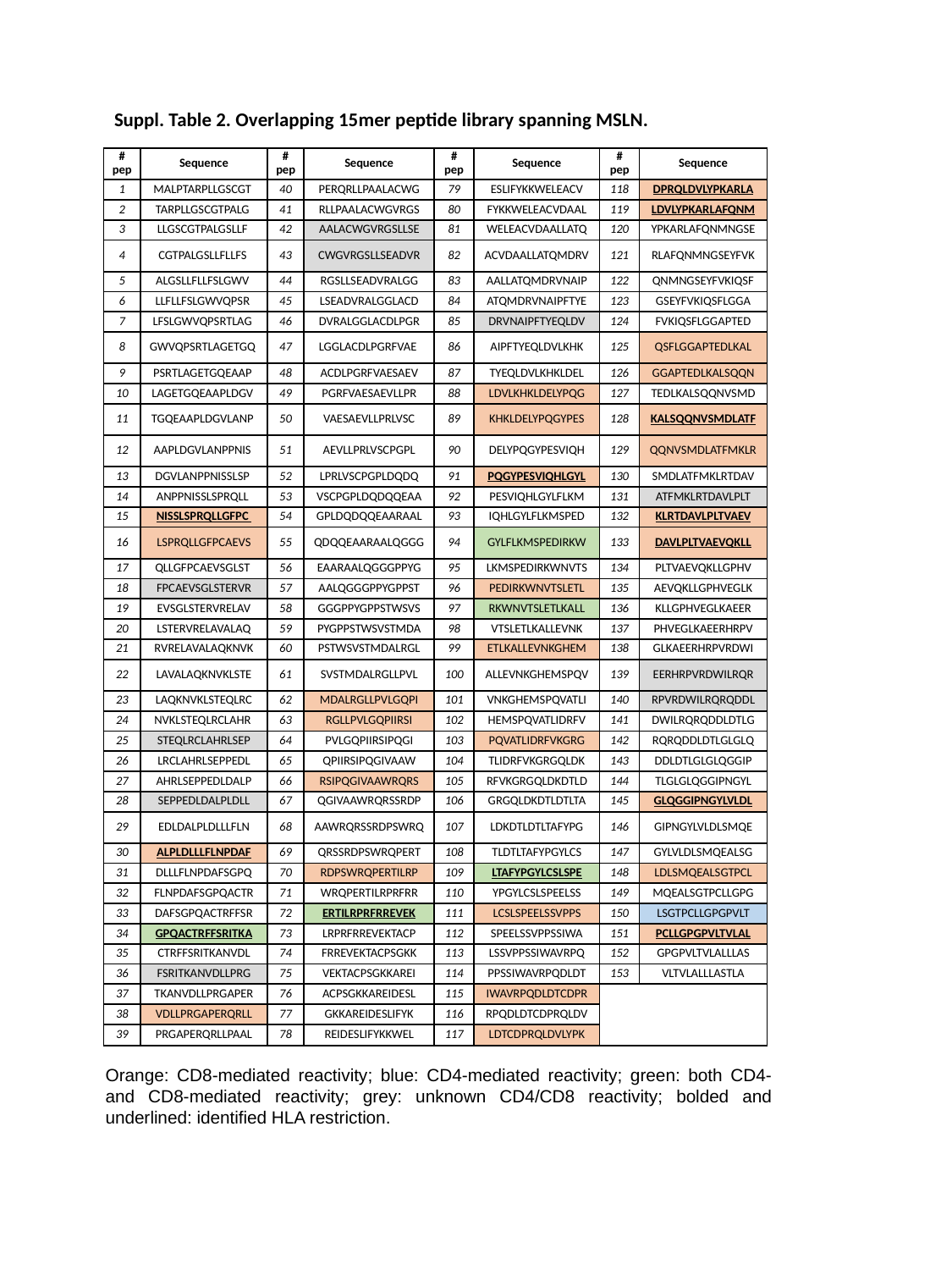

Suppl. Table 2. Overlapping 15mer peptide library spanning MSLN.
| # pep | Sequence | # pep | Sequence | # pep | Sequence | # pep | Sequence |
| --- | --- | --- | --- | --- | --- | --- | --- |
| 1 | MALPTARPLLGSCGT | 40 | PERQRLLPAALACWG | 79 | ESLIFYKKWELEACV | 118 | DPRQLDVLYPKARLA |
| 2 | TARPLLGSCGTPALG | 41 | RLLPAALACWGVRGS | 80 | FYKKWELEACVDAAL | 119 | LDVLYPKARLAFQNM |
| 3 | LLGSCGTPALGSLLF | 42 | AALACWGVRGSLLSE | 81 | WELEACVDAALLATQ | 120 | YPKARLAFQNMNGSE |
| 4 | CGTPALGSLLFLLFS | 43 | CWGVRGSLLSEADVR | 82 | ACVDAALLATQMDRV | 121 | RLAFQNMNGSEYFVK |
| 5 | ALGSLLFLLFSLGWV | 44 | RGSLLSEADVRALGG | 83 | AALLATQMDRVNAIP | 122 | QNMNGSEYFVKIQSF |
| 6 | LLFLLFSLGWVQPSR | 45 | LSEADVRALGGLACD | 84 | ATQMDRVNAIPFTYE | 123 | GSEYFVKIQSFLGGA |
| 7 | LFSLGWVQPSRTLAG | 46 | DVRALGGLACDLPGR | 85 | DRVNAIPFTYEQLDV | 124 | FVKIQSFLGGAPTED |
| 8 | GWVQPSRTLAGETGQ | 47 | LGGLACDLPGRFVAE | 86 | AIPFTYEQLDVLKHK | 125 | QSFLGGAPTEDLKAL |
| 9 | PSRTLAGETGQEAAP | 48 | ACDLPGRFVAESAEV | 87 | TYEQLDVLKHKLDEL | 126 | GGAPTEDLKALSQQN |
| 10 | LAGETGQEAAPLDGV | 49 | PGRFVAESAEVLLPR | 88 | LDVLKHKLDELYPQG | 127 | TEDLKALSQQNVSMD |
| 11 | TGQEAAPLDGVLANP | 50 | VAESAEVLLPRLVSC | 89 | KHKLDELYPQGYPES | 128 | KALSQQNVSMDLATF |
| 12 | AAPLDGVLANPPNIS | 51 | AEVLLPRLVSCPGPL | 90 | DELYPQGYPESVIQH | 129 | QQNVSMDLATFMKLR |
| 13 | DGVLANPPNISSLSP | 52 | LPRLVSCPGPLDQDQ | 91 | PQGYPESVIQHLGYL | 130 | SMDLATFMKLRTDAV |
| 14 | ANPPNISSLSPRQLL | 53 | VSCPGPLDQDQQEAA | 92 | PESVIQHLGYLFLKM | 131 | ATFMKLRTDAVLPLT |
| 15 | NISSLSPRQLLGFPC | 54 | GPLDQDQQEAARAAL | 93 | IQHLGYLFLKMSPED | 132 | KLRTDAVLPLTVAEV |
| 16 | LSPRQLLGFPCAEVS | 55 | QDQQEAARAALQGGG | 94 | GYLFLKMSPEDIRKW | 133 | DAVLPLTVAEVQKLL |
| 17 | QLLGFPCAEVSGLST | 56 | EAARAALQGGGPPYG | 95 | LKMSPEDIRKWNVTS | 134 | PLTVAEVQKLLGPHV |
| 18 | FPCAEVSGLSTERVR | 57 | AALQGGGPPYGPPST | 96 | PEDIRKWNVTSLETL | 135 | AEVQKLLGPHVEGLK |
| 19 | EVSGLSTERVRELAV | 58 | GGGPPYGPPSTWSVS | 97 | RKWNVTSLETLKALL | 136 | KLLGPHVEGLKAEER |
| 20 | LSTERVRELAVALAQ | 59 | PYGPPSTWSVSTMDA | 98 | VTSLETLKALLEVNK | 137 | PHVEGLKAEERHRPV |
| 21 | RVRELAVALAQKNVK | 60 | PSTWSVSTMDALRGL | 99 | ETLKALLEVNKGHEM | 138 | GLKAEERHRPVRDWI |
| 22 | LAVALAQKNVKLSTE | 61 | SVSTMDALRGLLPVL | 100 | ALLEVNKGHEMSPQV | 139 | EERHRPVRDWILRQR |
| 23 | LAQKNVKLSTEQLRC | 62 | MDALRGLLPVLGQPI | 101 | VNKGHEMSPQVATLI | 140 | RPVRDWILRQRQDDL |
| 24 | NVKLSTEQLRCLAHR | 63 | RGLLPVLGQPIIRSI | 102 | HEMSPQVATLIDRFV | 141 | DWILRQRQDDLDTLG |
| 25 | STEQLRCLAHRLSEP | 64 | PVLGQPIIRSIPQGI | 103 | PQVATLIDRFVKGRG | 142 | RQRQDDLDTLGLGLQ |
| 26 | LRCLAHRLSEPPEDL | 65 | QPIIRSIPQGIVAAW | 104 | TLIDRFVKGRGQLDK | 143 | DDLDTLGLGLQGGIP |
| 27 | AHRLSEPPEDLDALP | 66 | RSIPQGIVAAWRQRS | 105 | RFVKGRGQLDKDTLD | 144 | TLGLGLQGGIPNGYL |
| 28 | SEPPEDLDALPLDLL | 67 | QGIVAAWRQRSSRDP | 106 | GRGQLDKDTLDTLTA | 145 | GLQGGIPNGYLVLDL |
| 29 | EDLDALPLDLLLFLN | 68 | AAWRQRSSRDPSWRQ | 107 | LDKDTLDTLTAFYPG | 146 | GIPNGYLVLDLSMQE |
| 30 | ALPLDLLLFLNPDAF | 69 | QRSSRDPSWRQPERT | 108 | TLDTLTAFYPGYLCS | 147 | GYLVLDLSMQEALSG |
| 31 | DLLLFLNPDAFSGPQ | 70 | RDPSWRQPERTILRP | 109 | LTAFYPGYLCSLSPE | 148 | LDLSMQEALSGTPCL |
| 32 | FLNPDAFSGPQACTR | 71 | WRQPERTILRPRFRR | 110 | YPGYLCSLSPEELSS | 149 | MQEALSGTPCLLGPG |
| 33 | DAFSGPQACTRFFSR | 72 | ERTILRPRFRREVEK | 111 | LCSLSPEELSSVPPS | 150 | LSGTPCLLGPGPVLT |
| 34 | GPQACTRFFSRITKA | 73 | LRPRFRREVEKTACP | 112 | SPEELSSVPPSSIWA | 151 | PCLLGPGPVLTVLAL |
| 35 | CTRFFSRITKANVDL | 74 | FRREVEKTACPSGKK | 113 | LSSVPPSSIWAVRPQ | 152 | GPGPVLTVLALLLAS |
| 36 | FSRITKANVDLLPRG | 75 | VEKTACPSGKKAREI | 114 | PPSSIWAVRPQDLDT | 153 | VLTVLALLLASTLA |
| 37 | TKANVDLLPRGAPER | 76 | ACPSGKKAREIDESL | 115 | IWAVRPQDLDTCDPR | | |
| 38 | VDLLPRGAPERQRLL | 77 | GKKAREIDESLIFYK | 116 | RPQDLDTCDPRQLDV | | |
| 39 | PRGAPERQRLLPAAL | 78 | REIDESLIFYKKWEL | 117 | LDTCDPRQLDVLYPK | | |
Orange: CD8-mediated reactivity; blue: CD4-mediated reactivity; green: both CD4- and CD8-mediated reactivity; grey: unknown CD4/CD8 reactivity; bolded and underlined: identified HLA restriction.

## Slide 3
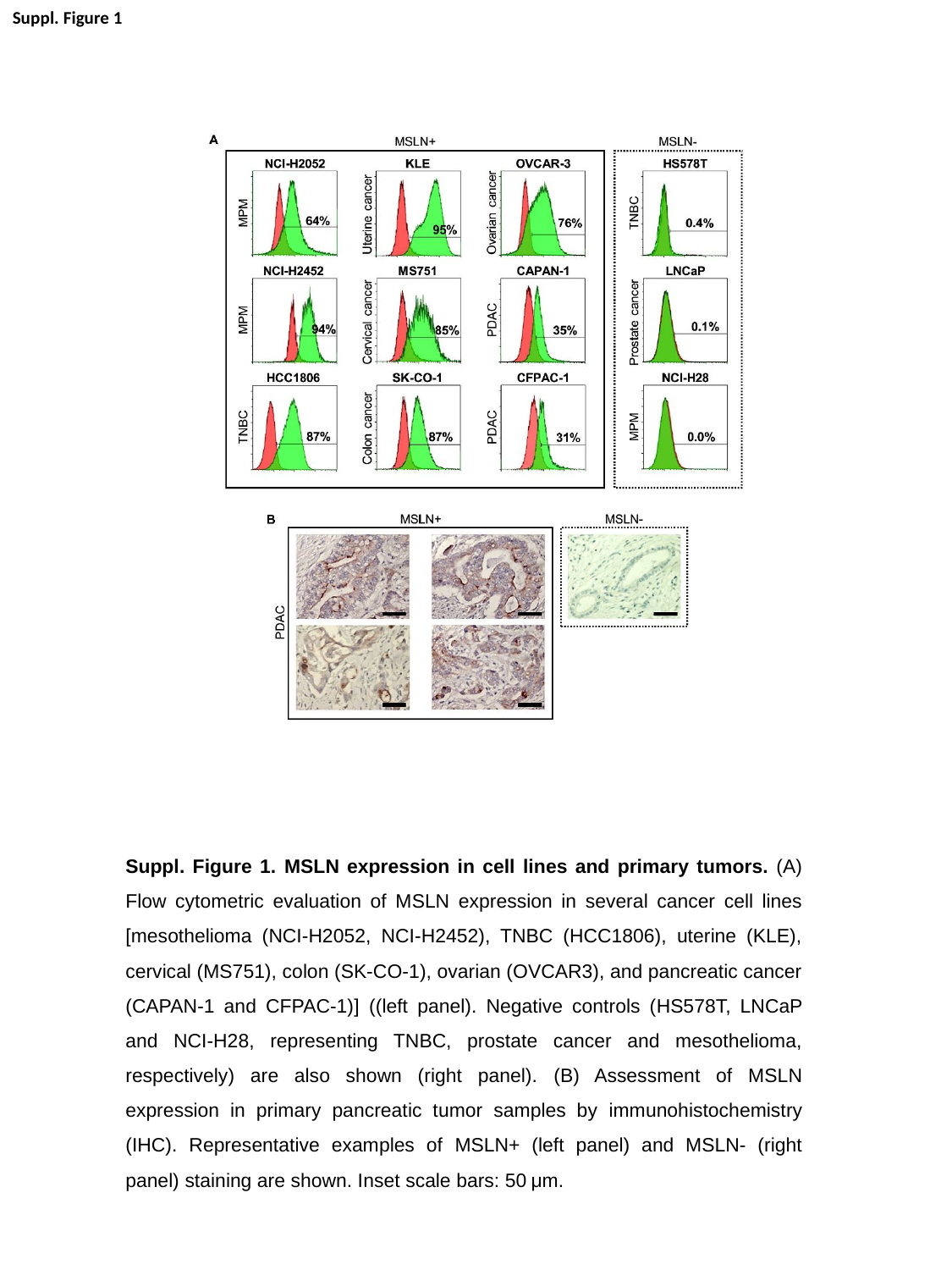

Suppl. Figure 1
Suppl. Figure 1. MSLN expression in cell lines and primary tumors. (A) Flow cytometric evaluation of MSLN expression in several cancer cell lines [mesothelioma (NCI-H2052, NCI-H2452), TNBC (HCC1806), uterine (KLE), cervical (MS751), colon (SK-CO-1), ovarian (OVCAR3), and pancreatic cancer (CAPAN-1 and CFPAC-1)] ((left panel). Negative controls (HS578T, LNCaP and NCI-H28, representing TNBC, prostate cancer and mesothelioma, respectively) are also shown (right panel). (B) Assessment of MSLN expression in primary pancreatic tumor samples by immunohistochemistry (IHC). Representative examples of MSLN+ (left panel) and MSLN- (right panel) staining are shown. Inset scale bars: 50 μm.

## Slide 4
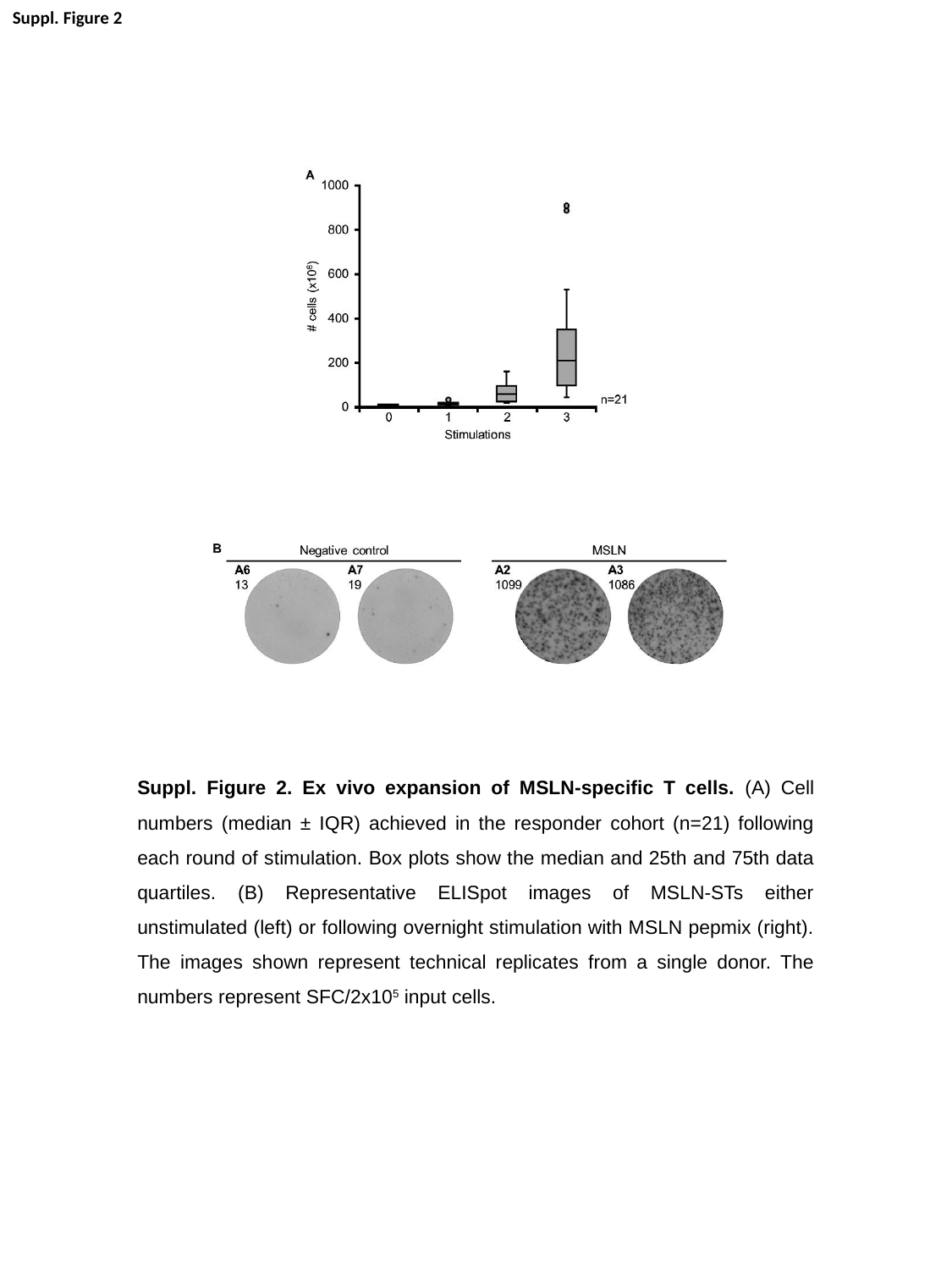

Suppl. Figure 2
### Chart
| Category | |
|---|---|
| 0 | 8.047619047619047 |
| 1 | 15.083333333333334 |
| 2 | 64.60619047619048 |
| 3 | 276.11857142857144 |
Suppl. Figure 2. Ex vivo expansion of MSLN-specific T cells. (A) Cell numbers (median ± IQR) achieved in the responder cohort (n=21) following each round of stimulation. Box plots show the median and 25th and 75th data quartiles. (B) Representative ELISpot images of MSLN-STs either unstimulated (left) or following overnight stimulation with MSLN pepmix (right). The images shown represent technical replicates from a single donor. The numbers represent SFC/2x105 input cells.

## Slide 5
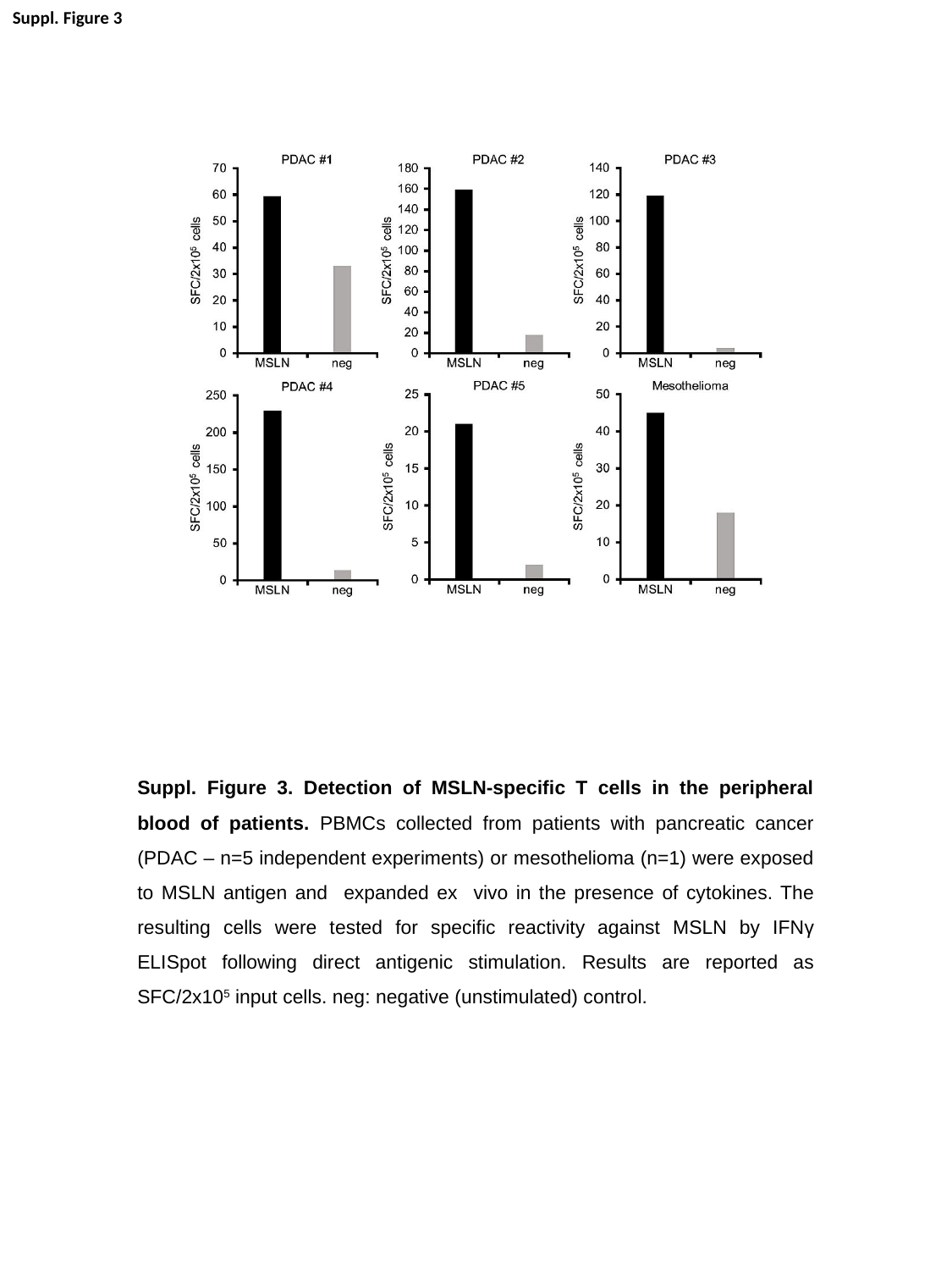

Suppl. Figure 3
Suppl. Figure 3. Detection of MSLN-specific T cells in the peripheral blood of patients. PBMCs collected from patients with pancreatic cancer (PDAC – n=5 independent experiments) or mesothelioma (n=1) were exposed to MSLN antigen and expanded ex vivo in the presence of cytokines. The resulting cells were tested for specific reactivity against MSLN by IFNγ ELISpot following direct antigenic stimulation. Results are reported as SFC/2x105 input cells. neg: negative (unstimulated) control.

## Slide 6
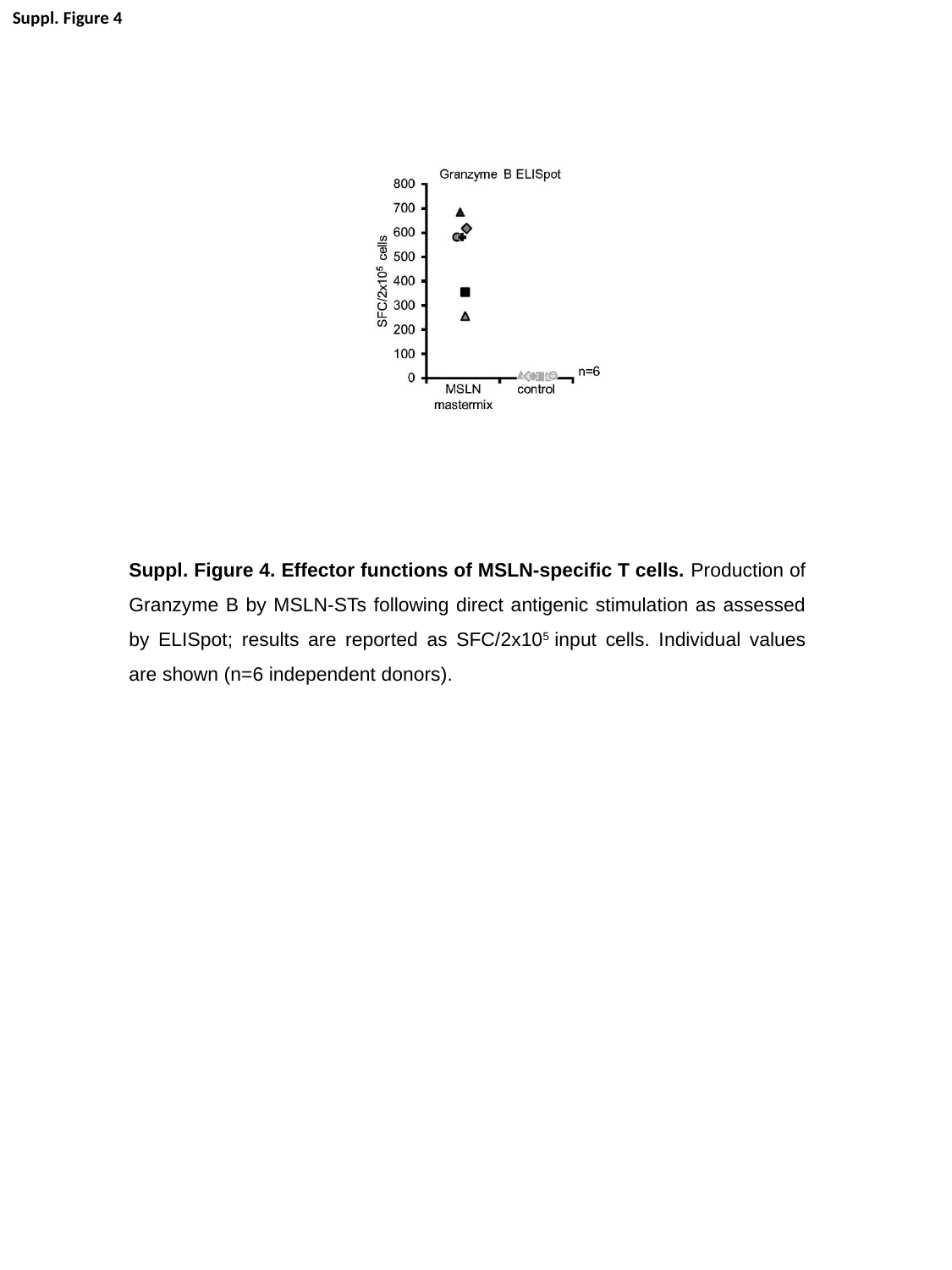

Suppl. Figure 4
Suppl. Figure 4. Effector functions of MSLN-specific T cells. Production of Granzyme B by MSLN-STs following direct antigenic stimulation as assessed by ELISpot; results are reported as SFC/2x105 input cells. Individual values are shown (n=6 independent donors).

## Slide 7
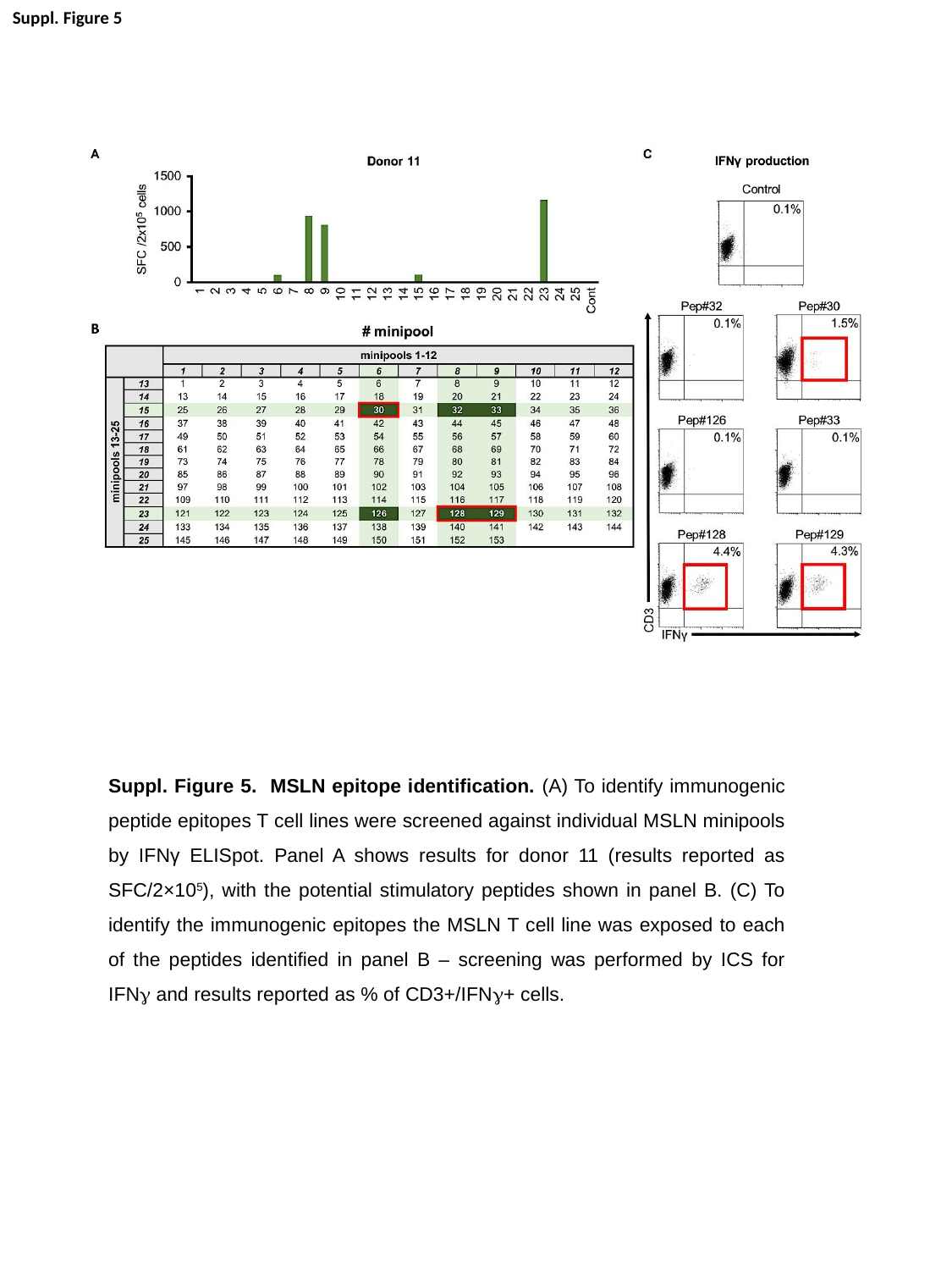

Suppl. Figure 5
Suppl. Figure 5. MSLN epitope identification. (A) To identify immunogenic peptide epitopes T cell lines were screened against individual MSLN minipools by IFNγ ELISpot. Panel A shows results for donor 11 (results reported as SFC/2×105), with the potential stimulatory peptides shown in panel B. (C) To identify the immunogenic epitopes the MSLN T cell line was exposed to each of the peptides identified in panel B – screening was performed by ICS for IFNγ and results reported as % of CD3+/IFNγ+ cells.

## Slide 8
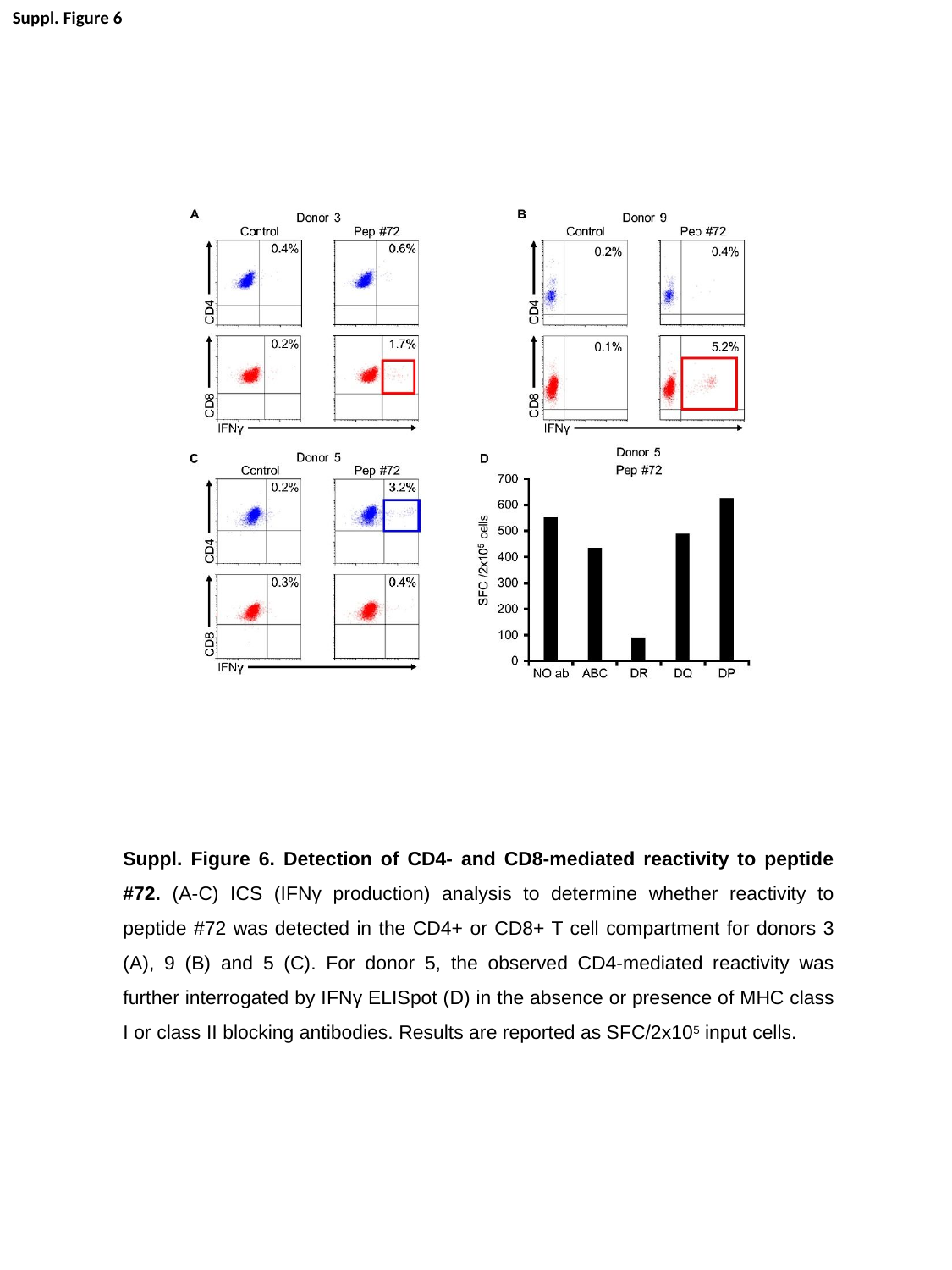

Suppl. Figure 6
Suppl. Figure 6. Detection of CD4- and CD8-mediated reactivity to peptide #72. (A-C) ICS (IFNγ production) analysis to determine whether reactivity to peptide #72 was detected in the CD4+ or CD8+ T cell compartment for donors 3 (A), 9 (B) and 5 (C). For donor 5, the observed CD4-mediated reactivity was further interrogated by IFNγ ELISpot (D) in the absence or presence of MHC class I or class II blocking antibodies. Results are reported as SFC/2x105 input cells.

## Slide 9
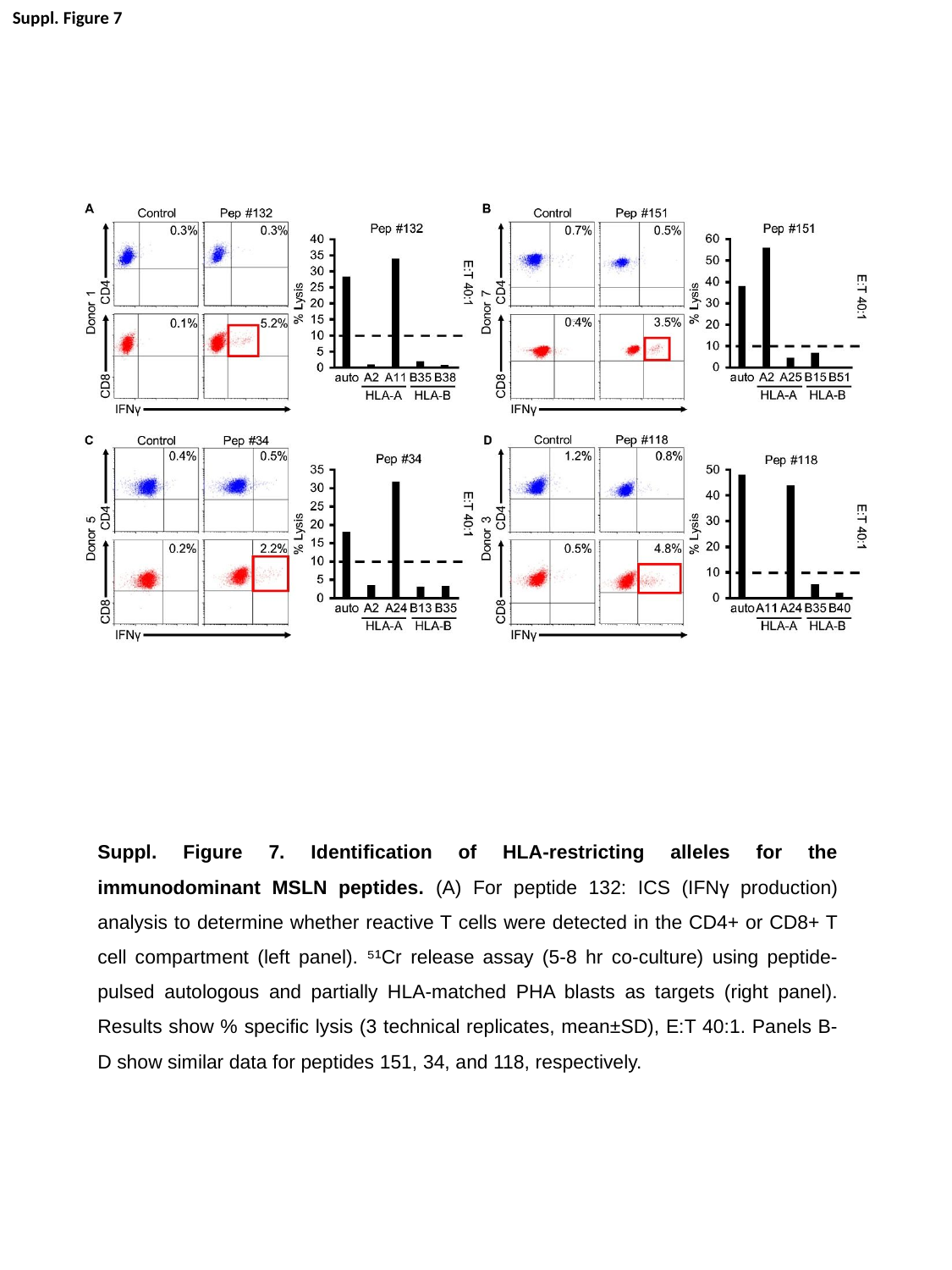

Suppl. Figure 7
Suppl. Figure 7. Identification of HLA-restricting alleles for the immunodominant MSLN peptides. (A) For peptide 132: ICS (IFNγ production) analysis to determine whether reactive T cells were detected in the CD4+ or CD8+ T cell compartment (left panel). ⁵¹Cr release assay (5-8 hr co-culture) using peptide-pulsed autologous and partially HLA-matched PHA blasts as targets (right panel). Results show % specific lysis (3 technical replicates, mean±SD), E:T 40:1. Panels B-D show similar data for peptides 151, 34, and 118, respectively.

## Slide 10
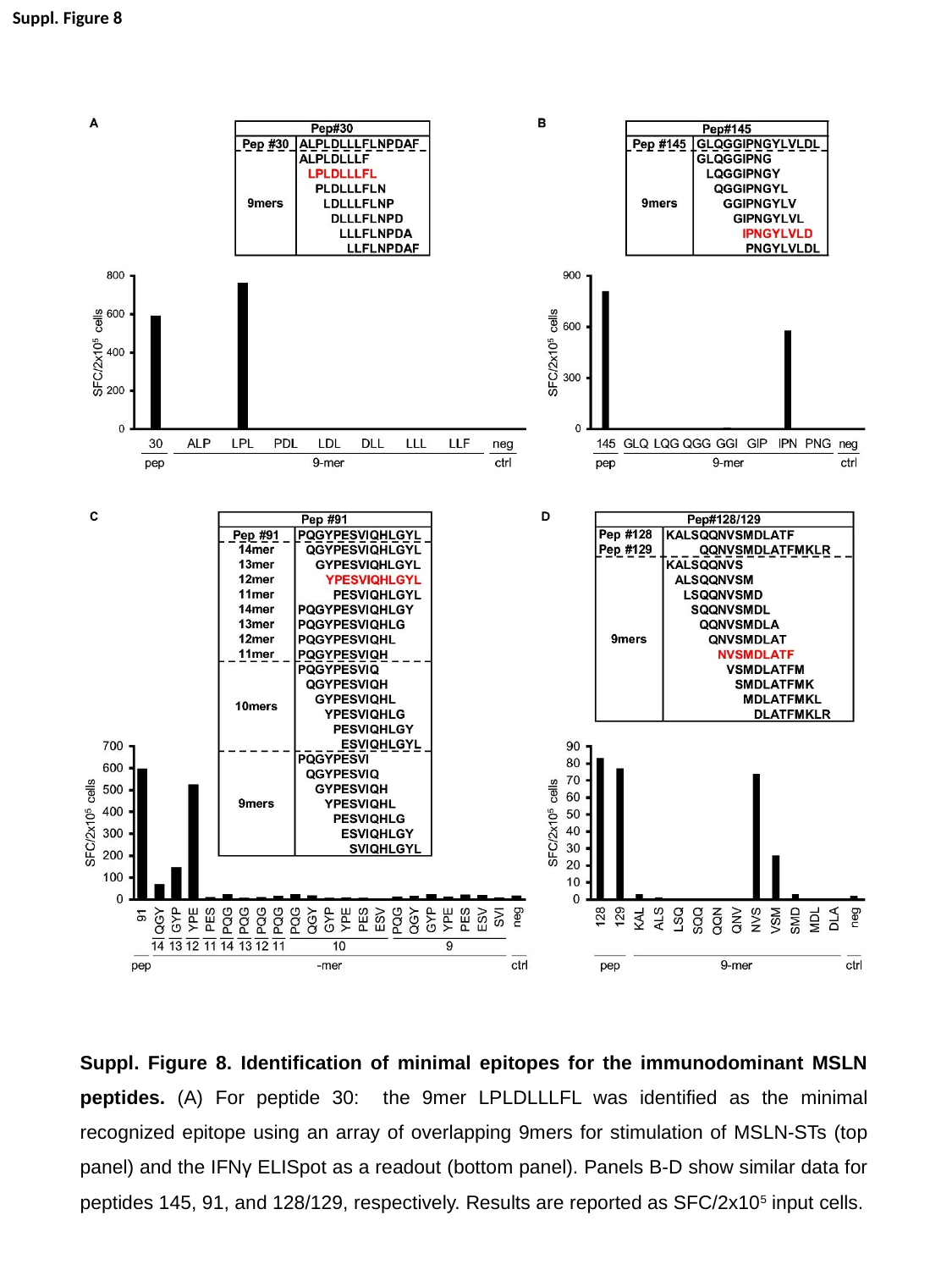

Suppl. Figure 8
Suppl. Figure 8. Identification of minimal epitopes for the immunodominant MSLN peptides. (A) For peptide 30: the 9mer LPLDLLLFL was identified as the minimal recognized epitope using an array of overlapping 9mers for stimulation of MSLN-STs (top panel) and the IFNγ ELISpot as a readout (bottom panel). Panels B-D show similar data for peptides 145, 91, and 128/129, respectively. Results are reported as SFC/2x105 input cells.
